# Plant Secondary Compounds Promote White Adipose Tissue Browning via Modulation of the Gut Microbiota in Small Mammals

**DOI:** 10.3390/ijms242417420

**Published:** 2023-12-13

**Authors:** Shien Ren, Liangzhi Zhang, Xianjiang Tang, Chao Fan, Yaqi Zhao, Qi Cheng, Yanming Zhang

**Affiliations:** 1 Key Laboratory of Adaptation and Evolution of Plateau Biota, Northwest Institute of Plateau Biology, Chinese Academy of Sciences, Xining 810008, China; 2 Qinghai Provincial Key Laboratory of Animal Ecological Genomics, Xining 810008, China; 3 University of Chinese Academy of Sciences, Beijing 100049, China

**Keywords:** plant secondary compounds, swainsonine, gut microbiota, white adipose tissue, brown adipose tissue

## Abstract

The browning of white adipose tissue (WAT) is a promising area of research for treating metabolic disorders and obesity in the future. However, studies on plant secondary compounds promoting WAT browning are limited. Herein, we explored the effects of swainsonine (SW) on gut microbiota and WAT browning in captive pikas. SW inhibited body mass gain, increased brown adipose tissue (BAT) mass, and induced WAT browning in pikas. The 16S rDNA sequencing revealed a significant reduction in the alpha diversity and altered community structure of the gut microbiota in captive pikas. However, the addition of SW to the diet significantly increased the alpha diversity of gut microbiota and the relative abundance of *Akkermansia*, *Prevotella*, and *unclassified_f__Lachnospiraceae*, along with the complexity of the microbial co-occurrence network structure, which decreased in the guts of captive pikas. Functional profiles showed that SW significantly decreased the relative abundances of energy metabolism, lipid metabolism, and glycan biosynthesis and metabolism, which were enriched in captive pikas. Furthermore, SW decreased deterministic processes of gut microbiota assembly in July and increased them in November. Finally, the genera *Prevotella* and *unclassified_f__Prevotellaceae* were positively correlated with BAT mass. Our results highlighted that plant secondary compounds promote WAT browning by modulating the gut microbiota in small mammals.

## 1. Introduction

Obesity poses a considerable threat to human health, leading to conditions such as hypertension and diabetes [1,2,3]. Statistics indicate that 2.1 billion people worldwide are overweight or obese, representing approximately 30% of the total population [4], and 4 million people die each year from obesity and related disorders [5]. Their treatment places strain on medical resources and carries a substantial economic burden [6]. In 2008, the total health care costs attributable to overweight and obesity in the United States were estimated to be as high as USD 147 billion [7]. The McKinsey Global Institute indicated that obesity causes a total economic impact of approximately USD 2 trillion per year, accounting for 2.8% of the world’s gross domestic product [8]. Obesity is a metabolic disorder syndrome due to overnutrition. Nonshivering thermogenesis, which occurs primarily in brown adipose tissue (BAT), can consume excess energy to re-establish the body’s energy balance [9]. Thus, the browning of white adipose tissue (WAT) is the most promising area of research for treating metabolic disorders and obesity in future [10,11,12].

Researchers have found that WAT can be converted into BAT upon cold exposure by increasing the expression of genes UCP1, UQCRC1, LETM1, and PGC-1α [13,14,15]. Moreover, intermittent fasting induces WAT browning by modulating the gut microbiota to elevate concentrations of the fermentation products acetate and lactate [16]. Plant secondary compounds (PSCs) induce WAT browning through significantly enhancing the activity of brown fat-specific genes FGF21, CIDEA, TMEM26, and TBX1 [17]. WAT browning contributes to reduced body fat accumulation and enhanced energy expenditure, and thus has potential therapeutic implications for the treatment of obesity [18]. The intestinal tract is the primary site where animals and humans digest food and absorb nutrients, and is a habitat for microorganisms. The gut microbiota plays an important role in regulating host nutrient metabolism, fat development and energy storage, and can promote fat accumulation via polysaccharide hydrolysis, fermentation and an increased production of short-chain fatty acids (SCFA) [19,20,21]. Therefore, it is necessary to investigate whether the browning effects of PSCs on WAT are mediated by gut microbiota.

Plateau pikas (*Ochotona curzoniae*), small mammals distributed on and adjacent to the Qinghai-Tibetan Plateau, exhibit a strong adaptation to low temperatures and oxygen levels [22,23,24]. Pikas maintain relatively stable body weight and body fat content, with no evident seasonal variation, and their BAT remains well developed throughout the year [25]. Swainsonine (SW) is an alkaloid secondary compound abundantly found in *Oxytropis* spp., which can cause poisoning and fatal outcomes in livestock, earning *Oxytropis* spp. the nickname “locoweeds” [26]. Despite the toxicity of SW in plants such as *Oxytropis kansuensis* and *Oxytropis ochrocephala*, plateau pikas favor these plants [27], indicating the strong adaptive ability of the species to the SW contained in these plants. Our previous studies found that SW remodels gut microbiota, promotes the rebounding of enterotypes, and maintains the stability of gut microbiota structure and function in pikas [28,29]. However, whether SW present in *Oxytropis* plants can further promote WAT browning by altering the structure and function of the gut microbiota of pikas remains unknown.

In this study, plateau pikas were used as an animal model to explore the effects of PSCs on the gut microbiota and WAT browning by adding SW to their diet. We identified the key gut bacteria involved in the regulation of WAT browning and provided a basic theoretical basis for the treatment of obesity and metabolic syndrome in humans.

## 2. Results

### 2.1. Changes in the Body Mass and BAT of Pikas

**Figure 1:**
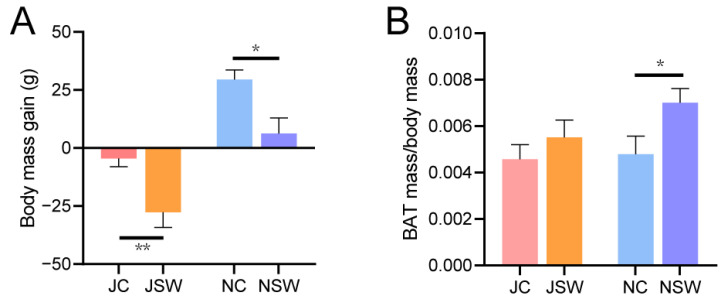
Effect of swainsonine (SW) on body mass and brown adipose tissue (BAT) mass in pikas. (**A**) Body mass gain and (**B**) BAT mass/body mass. * *p* < 0.05, ** *p* < 0.01.

**Figure 2:**
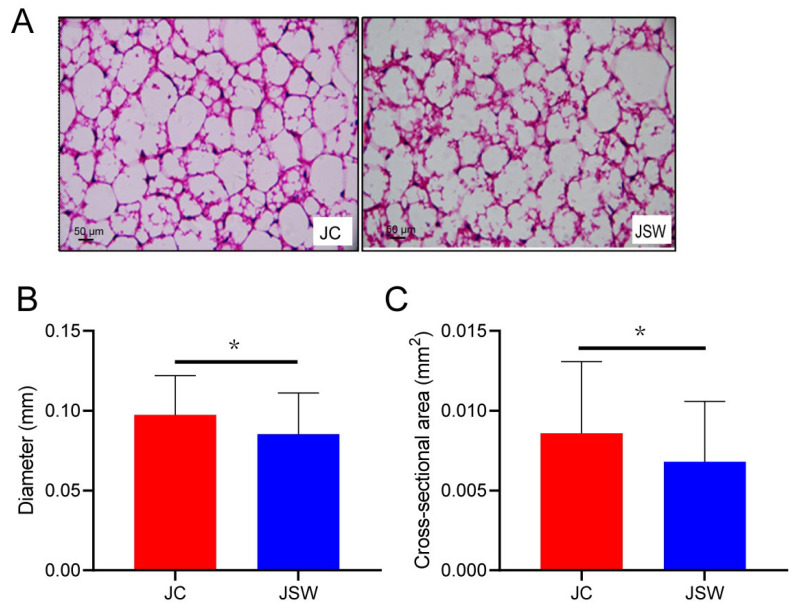
Swainsonine (SW) promotes white adipose tissue (WAT) browning in pikas. (**A**) The morphology of WAT cells. Scale bars = 50 μm. (**B**) Diameter and (**C**) cross-sectional area of WAT cells. * *p* < 0.05.

During the experiment, the body mass of pikas in the July captive (JC) and November SW-diet (NSW) groups showed no significant difference at the initial stage and the end of each stage. In contrast, the July SW-diet (JSW) group exhibited significantly lower body mass at the end than at the initial point of this stage, whereas the NC group demonstrated a significantly higher increase in body mass at the end than at the initial point of this stage (*p* < 0.01) (Figure S1A). The body mass gain of pikas was significantly higher in the JC group than in the JSW group, and in the November captive (NC) group than in the NSW group (*p* < 0.05) (Figure 1A). Although BAT mass did not differ significantly between the JC and JSW groups or between the NC and NSW groups (Figure S1B), the BAT mass/body mass ratio was significantly higher in the NSW group than in the NC group (*p* < 0.05) (Figure 1B). Ultra-structurally, the WAT cells of pikas in the JC group were relatively large, whereas those in the JSW group were relatively small, had smaller lipid droplets, became darker in color, and exhibited browning characteristics (Figure 2A). The statistical results also showed that the cell diameter and cross-sectional area of the WAT in the JSW group were significantly smaller than those in the JC group (*p* < 0.05) (Figure 2B,C).

### 2.2. The Differences in the Gut Microbiota Diversity of Pikas

**Figure 3:**
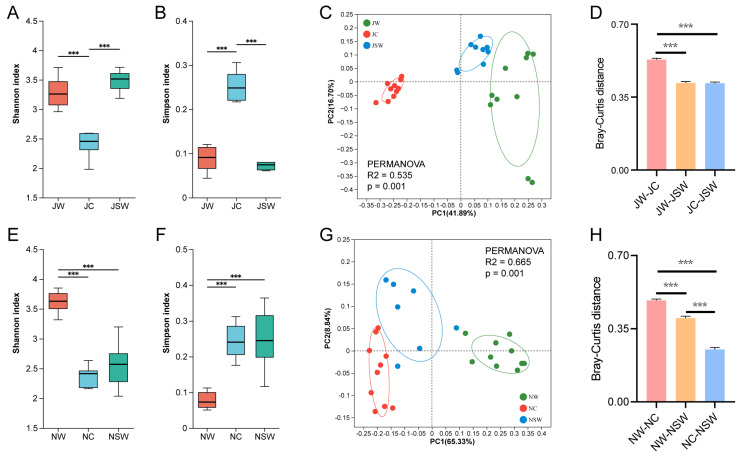
Changes in gut microbial diversity in pikas. (**A**,**E**) Shannon index, (**B**,**F**) Simpson index, (**C**,**G**) Principal coordinate analysis (PCoA) based on the Bray–Curtis distances. Ellipses imply 95% confidence intervals for each group. (**D**,**H**) Differences in Bray–Curtis distances in July and November. *** *p* < 0.001.

In July, the Shannon index of the gut microbiota of pikas was significantly higher, and the Simpson index was significantly lower in the July wild (JW) and JSW groups than that in the JC group (*p* < 0.001) (Figure 3A,B). A principal coordinate analysis (PCoA) based on the Bray–Curtis distance showed significant differences in bacterial communities among the three groups (permutational multivariate analysis of variance [PERMANOVA], R^2^ = 0.535, *p* = 0.001) (Figure 3C). Moreover, the pairwise analysis revealed significant differences between any two groups (PERMANOVA: JW vs. JC, R^2^ = 0.587, *p* = 0.001; JW vs. JSW, R^2^ = 0.299, *p* = 0.001; JC vs. JSW, R^2^ = 0.598, *p* = 0.001) (Table S1). In addition, the Bray–Curtis distance between the JW and JC groups was significantly longer than that between the JW and JSW groups and between the JC and JSW groups (*p* < 0.001) (Figure 3D).

In November, the Shannon index of the gut microbiota of pikas in the November wild (NW) group was significantly higher than that in the NC and NSW groups, whereas the Simpson index was significantly lower than that in the NC and NSW groups (*p* < 0.001) (Figure 3E,F). A PCoA based on the Bray–Curtis distance showed significant differences in bacterial communities among the three groups (PERMANOVA, R^2^ = 0.665, *p* = 0.001) (Figure 3G). Furthermore, the pairwise analysis indicated significant differences between any two groups (PERMANOVA: NW vs. NC, R^2^ = 0.699, *p* = 0.001; NW vs. NSW, R^2^ = 0.554, *p* = 0.001; NC vs. NSW, R^2^ = 0.297, *p* = 0.001) (Table S1). In addition, the Bray–Curtis distance between the NW and NC groups was significantly longer than that between the NW and NSW groups and between the NC and NSW groups, which was significantly longer than that between the NC and NSW groups (*p* < 0.001) (Figure 3H).

### 2.3. The Composition and Differences in the Gut Microbiota of Pikas

**Figure 4:**
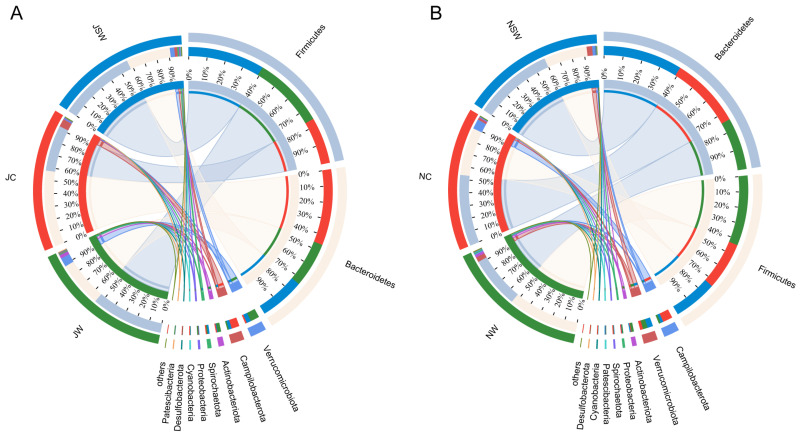
Gut microbiota composition in pikas. Pika gut bacteria composition at the phylum level in (**A**) July and (**B**) November.

**Figure 5:**
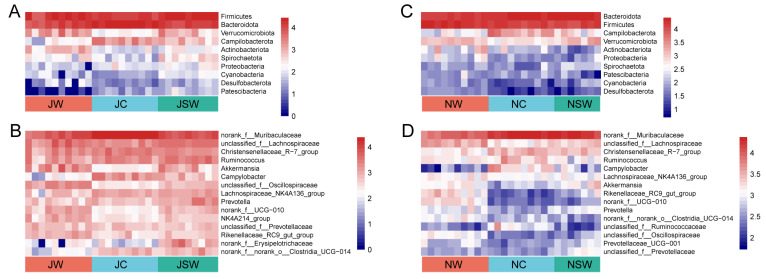
Gut microbiota changes in pikas. Gut microbiota changes at the (**A**,**C**) phylum and (**B**,**D**) genus levels.

In July, the dominant bacteria in the gut microbiota of pikas at the phylum level were Firmicutes (48.04%), Bacteroidetes (42.75%), and Verrucomicrobiota (3.62%) (Figure 4A). The dominant bacteria at the genus level were *norank _f__Muribaculaceae* (32.17%), *unclassified_f__Lachnospiraceae* (8.52%), *Christensenellaceae_R−7_group* (6.31%), *Ruminococcus* (5.75%), and *Akkermansia* (3.62%) (Figure S2). The statistical analysis indicated that eight bacterial phyla were significantly different between the JW and JC groups, among which the relative abundances of Firmicutes, Verrucomicrobiota, Actinobacteriota, Spirochaetota, and Cyanobacteria were significantly higher in the JW group than in the JC group, whereas the relative abundances of Bacteroidetes, Campylobacteroides, and Patescibacteria were significantly lower in the JW group than in the JC group (*p* < 0.05). Four bacterial phyla were significantly different between the JW and JSW groups, specifically, the relative abundance of Actinobacteriota in the JW group was significantly higher than that in the JSW group, whereas the relative abundances of Campilobacterota, Proteobacteria, and Patescibacteria were significantly lower than that in the JSW group (*p* < 0.05) (Figure 5A and Table S2). At the genus level, 10 bacterial genera were significantly different between the JW and JC groups, among which *unclassified_f__Lachnospiraceae*, *Akkermansia*, *unclassified_f__Oscillospiraceae*, *norank_f__UCG−010*, *NK4A214_group*, and *Rikenellaceae_RC9_gut_group* were significantly enriched in the JW group, whereas the *norank_f__Muribaculaceae*, *Campylobacter*, *Lachnospiraceae_NK4A136_group*, and *norank_f__norank_o__Clostridia_UCG−014* were significantly enriched in the JC group (*p* < 0.01). Nine bacterial genera were significantly different between the JW and JSW groups and *unclassified_f__Oscillospiraceae*, *norank_f__UCG−010*, *NK4A214_group*, and *Rikenellaceae_RC9_gut_group* were enriched in the JW group, whereas *Ruminococcus*, *Campylobacter*, *Lachnospiraceae_NK4A136_group*, *Prevotella*, and *norank_f__norank_o__Clostridia_UCG−014* were enriched in the JSW group (*p* < 0.05) (Figure 5B and Table S2).

In November, the dominant bacteria in gut microbiota at the phylum level were Firmicutes (41.40%), Bacteroidetes (49.86%), and Campilobacterota (3.61%) (Figure 4B). The dominant bacteria at the genus level were *norank _f__Muribaculaceae* (39.78%), *unclassified_f__Lachnospiraceae* (7.08%), *Christensenellaceae_R−7_group* (6.51%), *Ruminococcus* (4.95%), and *Lachnospiraceae_NK4A136_group* (3.36%) (Figure S3). Furthermore, seven bacterial phyla were significantly different between the NW and NC groups, among which the relative abundances of Firmicutes, Verrucomicrobiota, Proteobacteria, Cyanobacteria, and Desulfobacterota were significantly higher in the NW group than those in the NC group, whereas the relative abundances of Bacteroidetes and Campilobacterota were significantly lower in the NW group than in the NC group (*p* < 0.01). Four bacterial phyla were significantly different between the NW and NSW groups, specifically, the relative abundances of Firmicutes and Actinobacteriota. Proteobacteria were significantly higher in the NW group than that in the NSW group, whereas the relative abundance of Bacteroidetes was significantly lower than that in the NSW group (*p* < 0.05) (Figure 5C and Table S3). At the genus level, 13 bacterial genera were significantly different between the NW and NC groups, among which *unclassified_f__Lachnospiraceae*, *Lachnospiraceae_NK4A136_group*, *Akkermansia*, *Rikenellaceae_RC9_gut_group*, *norank_f__UCG−010*, *Prevotella*, *norank_f__norank_o__Clostridia_UCG−014*, *unclassified_f__Oscillospiraceae*, and *Prevotellaceae_UCG−001* were significantly enriched in the NW group. However, *norank_f__Muribaculaceae*, *Christensenellaceae_R−7_group*, *Campylobacter*, and *unclassified_f__Ruminococcaceae* were significantly enriched in the NC group (*p* < 0.05). Eight bacterial genera were significantly different between the NW and NSW groups, and *unclassified_f__Lachnospiraceae*, *Rikenellaceae_RC9_gut_group*, *norank_f__UCG−010*, *norank_f__norank_o__Clostridia_UCG−014*, and *unclassified_f__Oscillospiraceae* were enriched in the NW group, whereas *norank_f__Muribaculaceae*, *Christensenellaceae_R−7_group*, and *unclassified_f__Prevotellaceae* were enriched in the NSW group (*p* < 0.01) (Figure 5D and Table S3).

### 2.4. The Co-Occurrence Network of Gut Microbiota in Pikas

**Figure 6:**
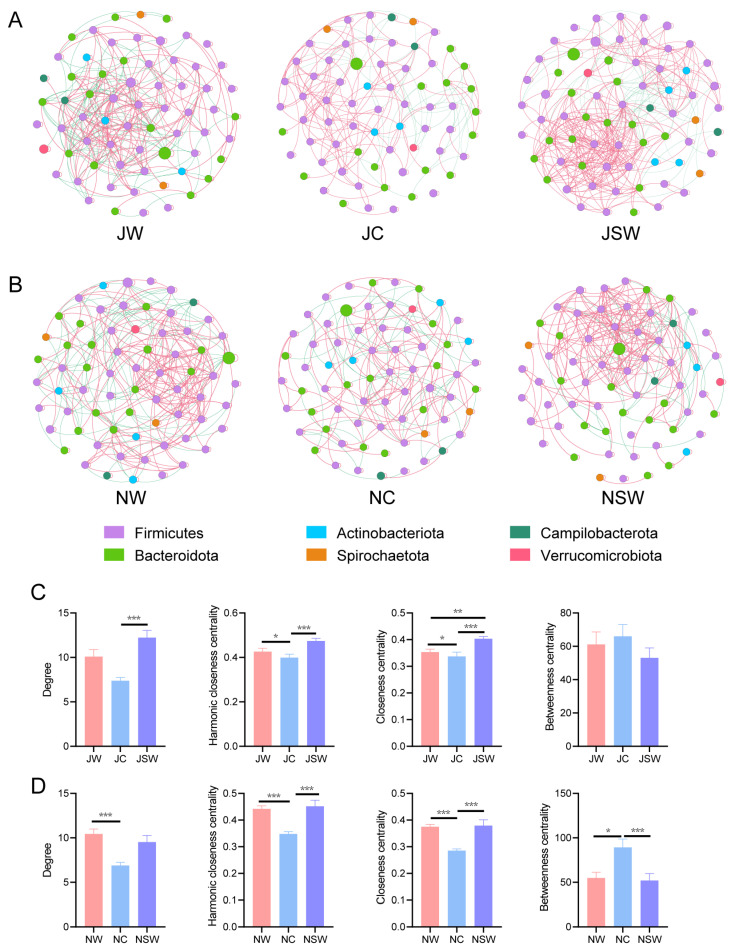
Co-occurrence network of the gut microbiota in pikas. Co-occurrence network of pika gut microbiota in (**A**) July and (**B**) November. Node-level topological features of the co-occurrence network in (**C**) July and (**D**) November. * *p* < 0.05, ** *p* < 0.01, *** *p* < 0.001.

Co-occurrence networks revealed interactions among the gut microbiota of pikas. In July, the JW group had 66 nodes and 333 links, the JC group had 68 nodes and 251 links, and the JSW group had 69 nodes and 422 links (Figure 6A and Table S4). We calculated the node-level topological parameters for each group. The results showed that the degree was significantly higher in the JSW group than that in the JC group (*p* < 0.001). The harmonic closeness centrality was significantly higher in the JW and JSW groups than that in the JC group (*p* < 0.05). Closeness centrality was significantly higher in the JW and JSW groups than that in the JC group, and in the JSW group than that in the JC group (*p* < 0.05). However, betweenness centrality was not significantly different between the groups (Figure 6C).

In November, there were 68 nodes and 355 links in the NW group, 69 nodes and 238 links in the NC group, and 69 nodes and 329 links in the NSW group (Figure 6B and Table S4). The node-level topological parameters showed that the degree was significantly higher in the NW group than that in the NC group (*p* < 0.001). Harmonic closeness centrality and closeness centrality were significantly higher in the NW and NSW groups than those in the NC group (*p* < 0.001). Betweenness centrality was significantly lower in the NW and NSW groups than that in the NC group (*p* < 0.05) (Figure 6D).

### 2.5. The Functional Profile of the Gut Microbiota in Pikas

**Figure 7:**
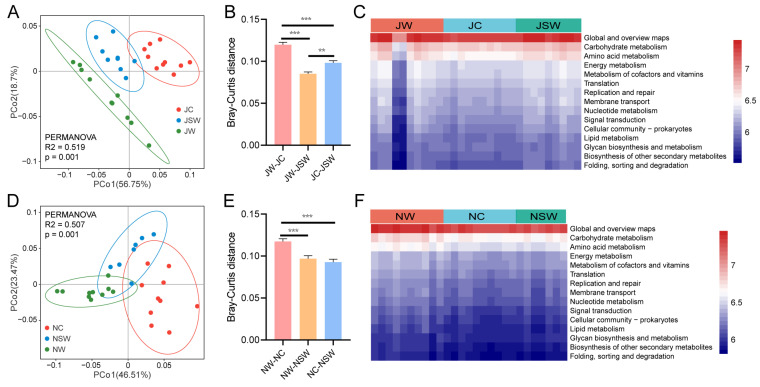
Changes in gut microbiota function in pikas. (**A**,**D**) PCoA based on the Bray–Curtis distances. Ellipses imply 95% confidence intervals for each group. (**B**,**E**) Differences in Bray–Curtis distances in July and November. (**C**,**F**) Changes in gut microbiota function at KEGG level 2. ** *p* < 0.01, *** *p* < 0.001.

A PCoA based on Bray–Curtis distance was used to display distinctions in the functional profiles of the gut microbiota in pikas. In July, the KEGG functional profiles differed significantly between the groups (PERMANOVA, R^2^ = 0.519, *p* = 0.001) (Figure 7A). A Pairwise analysis revealed significant differences between any two groups (PERMANOVA: JW vs. JC, R^2^ = 0.214, *p* = 0.002; JW vs. JSW, R^2^ = 0.248, *p* = 0.006; JC vs. JSW, R^2^ = 0.519, *p* = 0.002) (Table S5). Furthermore, the Bray–Curtis distance between the JW and JC groups and between the JC and JSW groups was significantly longer than that between the JW and JSW groups (*p* < 0.01) (Figure 7B). In particular, the heatmap showed that the abundances of the KEGG categories of energy metabolism, lipid metabolism, glycan biosynthesis and metabolism were significantly lower in the JW group than those in the JC group (*p* < 0.05); however, no significant differences were found in these metabolic categories between the JW and JSW groups (Figure 7C and Table S6).

In November, a PCoA revealed significant differences in the KEGG functional profiles between the groups (PERMANOVA, R^2^ = 0.507, *p* = 0.001) (Figure 7D). A Pairwise analysis identified significant differences between any two groups (PERMANOVA: NW vs. NC, R^2^ = 0.479, *p* = 0.001; NW vs. NSW, R^2^ = 0.401, *p* = 0.001; NC vs. NSW, R^2^ = 0.370, *p* = 0.001) (Table S5). Furthermore, the Bray–Curtis distance between the NW and NC groups was significantly greater than that between the NW and NSW groups, and between the NC and NSW groups (*p* < 0.001) (Figure 7E). Specifically, the heatmap showed that the abundance of the KEGG categories of global and overview maps, translation, folding, sorting, and degradation was significantly lower in the NW group than that in the NC group (*p* < 0.05); however, no significant differences were observed among these metabolic categories between the NW and NSW groups (Figure 7F and Table S7).

### 2.6. The Assembly of Gut Microbiota in Pikas

**Figure 8:**
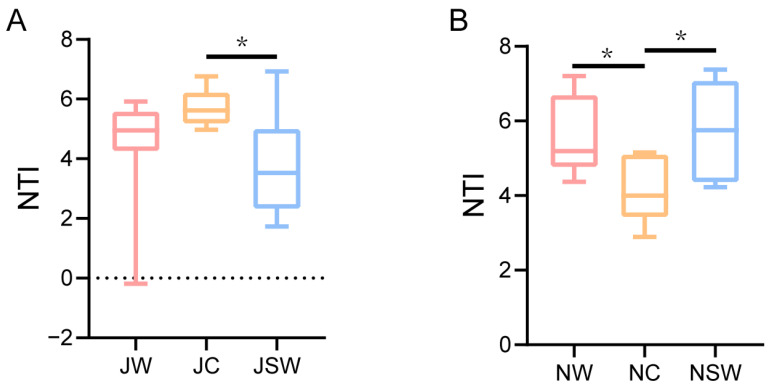
Assembly processes of gut microbiota in pikas. Assembly processes of gut microbiota of pikas in (**A**) July and (**B**) November. The dotted line represents a zero NTI value. * *p* < 0.05.

To explore the effects of SW on the assembly of gut microbiota in pikas, we calculated the nearest taxon index (NTI) of the gut bacteria in each group. The mean NTIs of all communities were significantly >0, indicating clustering within the sample group. When the NTIs of a community were >2, co-existing taxa were more closely related than expected by chance (phylogenetic clustering). In July, 97% of the NTIs were >0, and 90% were >2. In November, all the NTIs exceeded 2. This implied that a deterministic process predominantly controls the gut microbial assembly in pikas, surpassing the influence of a stochastic process. The results of the Kruskal–Wallis test showed that NTIs were significantly greater in the JC group than those in the JSW group (*p* < 0.05) (Figure 8A), and significantly greater in the NW and NSW groups than those in the NC group (*p* < 0.05) (Figure 8B).

### 2.7. The Relationship between BAT and Gut Microbiota

**Figure 9:**
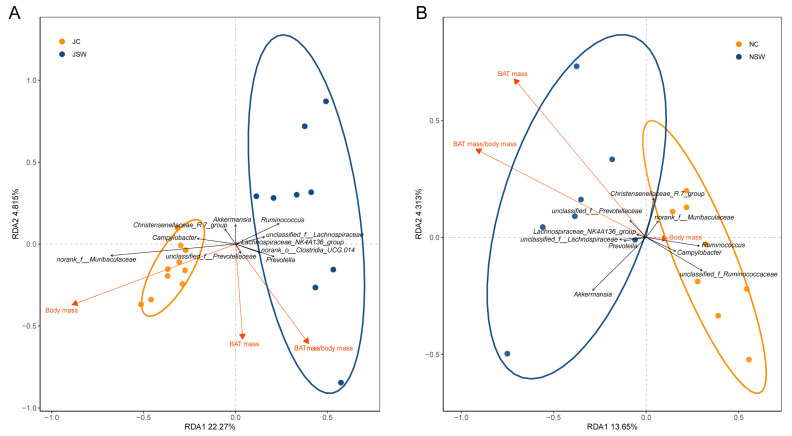
Redundancy analysis (RDA) plots of gut microbiota and host phenotype in pikas. Relationship between gut microbiota and host phenotype of pikas in (**A**) July and (**B**) November. Ellipses imply 95% confidence intervals for each group. Black arrows represent gut microbes and orange arrows indicate host phenotypes.

A redundancy analysis (RDA) was used to reveal the relationship between BAT and gut microbiota in plateau pikas. In July, there was no clear correlation between BAT mass and gut microbiota in the JC and JSW groups; however, BAT mass/body mass was positively correlated with the gut microbiota in the JSW group (Figure 9A). In November, BAT mass and BAT mass/body mass were positively correlated with the gut microbiota in the NSW group and negatively correlated with the gut microbiota in the NC group (Figure 9B).

## 3. Discussion

Our results showed that SW significantly reduced body mass gain, induced smaller WAT cells, and increased BAT mass in plateau pikas (Figure 1 and Figure 2). This implied that SW may prevent excessive host adiposity by promoting WAT browning. Natural products derived from plants have beneficial effects in preventing and treating obesity and its associated metabolic disorders [30,31,32]. For example, flavonoids inhibit body mass gain by reducing food intake and increasing satiety [33]. Carnosic acid (a diterpenoid), a major component of the labiate herbal plant, rosemary (Rosmarinus officinalis), has beneficial effects in combating obesity and type 2 diabetes [34]. The consumption of alkaloids such as caffeine, ephedrine, or capsaicin increases the lipolytic response and elevates the metabolic rate, thereby contributing to increased energy expenditure and body mass loss [35,36]. The body mass-loss effect of PSCs may be achieved by promoting WAT browning, thereby increasing host energy expenditure. WAT specializes in storing excess energy as triglycerides, whereas BAT primarily specializes in high metabolism and energy expenditure. Several PSCs induce WAT browning [18]. Luteolin, a natural flavonoid, activates WAT browning and thermogenesis via the AMPK/PGC1α pathway, preventing overweight and insulin resistance induced by a high-fat diet [37]. Resveratrol (a phenolic compound) promotes WAT browning by activating peroxisome proliferator-activated receptors, inducing the thermogenic capacity of interscapular BAT by increasing mitochondrial production, enhancing fatty acid oxidation and glucose disposal [38].

Captive conditions significantly reduced the alpha diversity of the gut microbiota in pikas, which was restored to the original wild state after feeding with SW (Figure 3A,B). Captivity induces gut microbiota dysbiosis in golden Guizhou snub-nosed monkeys (*Rhinopithecus brelichi*) [39], Tibetan wild asses (*Equus kiang*) [40], and forest musk deer (*Moschus berezovskii*) [41]. This dysbiosis could be attributed to marked changes in diet, with substantial changes in nutrient composition between artificial and natural diets. The simplicity of artificial diets may drive differences in the gut microbiota compared to the more complex natural diets [42]. Furthermore, artificial diets high in fat and protein often result in a decrease in gut microbiota diversity [43]. Recently, several studies have revealed that PSCs promote gut microbiota diversity [44]. Kohl et al. found that creosote toxins contained in the creosote bush (*Larrea tridentata*) can increase gut microbial diversity in woodrats (*Neotoma*) [45]. Phenolic compounds, while promoting the diversity of the gut microbiota, are also beneficial for maintaining colon health [44]. Higher gut microbiota diversity is beneficial for the host in managing external environmental fluctuations [46]. Our results implied that SW plays a crucial role in maintaining the intestinal microecological stability in small mammals.

Compared to the JW group, the JC group had a higher abundance of Bacteroidetes and a lower abundance of Firmicutes (Figure 5A). Bacteroidetes are associated with protein breakdown, and Firmicutes with cellulose degradation. The artificial diet contained higher protein and lower cellulose content than the wild diet, which may be responsible for the fluctuating abundances of Firmicutes and Bacteroidetes [42]. However, after the addition of SW to the artificial diet, the abundances of Firmicutes and Bacteroidetes in the pikas in the JSW group were not significantly different from those in the JW group. Similarly, dietary supplementation with resveratrol [47] and phenolic ingredients [48] decreased the abundance of Bacteroidetes and increased the abundance of Firmicutes in mice. After dietary supplementation with SW, the abundance of the probiotic bacteria *Akkermansia*, *Prevotella*, and *unclassified_f__Lachnospiraceae* returned to their original levels. Similar studies have found that puerarin significantly increases the abundance of *Akkermansia* in rat intestines [49]. Dietary supplementation with flavonoids increases *Prevotella* abundance in the calf intestine [50]. In vitro fermentation experiments have shown that thymol increases the abundance of *Lachnospiraceae* in the rumen [51]. This illustrates that the maintenance and suppression effects of SW on the gut microbial abundance are consistent with those of other PSCs.

The complexity of the co-occurrence network of the gut microbiota of pikas was reduced in captivity; however, it was restored to the wild state after the addition of SW to the artificial diet (Figure 6). Similarly, laboratory experiments found that Brandt’s voles (*Lasiopodomys brandtii*) harbored a more complex microbial network in the high-tannin group [52]. A complex network of gut microbiota may contribute to food degradation and nutrient extraction in herbivores, and reduce the toxicity of PSCs, which are abundant in their diet [52]. In vivo experiments have shown that the mixture of all cultured bacteria had a higher rate of tea saponin degradation than any single isolate [53]. Mixed cultures of *Methanobrevibacter* spp. and *Methanosphaera stadtmanae* from the hoatzin *Opisthocomus hoazin* were able to reduce the hemolytic activity of *Quillaja* saponins by 80% within a few hours [54]. Furthermore, complex microbial networks exhibit superior resilience against external disturbances compared to simple networks [55], which is beneficial for maintaining homeostasis in the host gut microecosystem.

The Bray–Curtis distance based on KEGG functional profiles showed that the JSW group was closer to the JW group than to the JC group, and the NSW group was closer to the NW group than to the NC group (Figure 7). This implied that SW can promote the recovery of gut microbiota function in pikas. This phenomenon can be explained by the consistency of changes in the composition and function of the gut microbiota [56]. Specifically, energy metabolism, lipid metabolism, and glycan biosynthesis and metabolism did not significantly differ between the JW and JSW groups. Changes in the gut microbiota function in pikas may be attributed to variations in dietary composition, as large differences exist between artificial and natural diets. Natural foods help to maintain the gut microbial function of white-throated woodrats (*Neotoma albigula*) better than artificial diets [57]. Our results suggested that PSCs in natural diets can prevent microbial dysfunction resulting from dietary differences.

The gut microbiota of captivity animals usually has strong deterministic processes compared to those of wild individuals [58]; however, our results suggest that deterministic processes in the gut microbiota of individuals under captivity are weakened compared to those of wild pikas (Figure 8B). Numerous studies have shown seasonal variations in the gut microbiota of wildlife [56,59,60], thus inconsistencies in sampling times may contribute to differences in the assembly of microbial communities. Moreover, SW decreased the deterministic process of gut microbiota assembly in July, whereas it increased it in November. The assembly process of gut microbiota in pikas can be complex, influenced by various environmental factors [61] and host physiological characteristics [62]. Therefore, the effects of SW on this process may be seasonally or duration-dependent on captivity.

A positive correlation between BAT mass and the genera *Prevotella* and *unclassified_f__Prevotellaceae* was observed both in July and November (Figure 9). These bacteria are important producers of SCFAs [63,64], which mediate a range of physiological functions such as signaling molecules in addition to being energy substances [65]. Acetate, in particular, has been implicated as an inducer of WAT browning by inducing mitochondrial biogenesis [66]. Resveratrol regulates bile acid metabolism by remodeling the gut microbiota, and lithocholic acid in bile acid metabolism can upregulate the uncoupling protein 1 expression by activating Takeda G-protein-coupled receptor 5, which induces WAT browning [67]. A similar study suggested that resveratrol-remodeled gut microbiota may induce WAT browning via sirtuin-1 signaling [47]. Our results highlighted the ability of SW to promote WAT browning by modulating the gut microbiota.

## 4. Materials and Methods

### 4.1. Experimental Animals

Sixty adult plateau pikas, thirty in July and thirty in November, were captured in Gangcha County using the rope-trap method. Thereafter, 10 pikas were randomly selected from July and November and were euthanized. After dissection on a sterile table, fecal samples were immediately collected in 2 mL cryovials, stored in a portable liquid nitrogen tank, transported to the laboratory, and stored in an ultra-low temperature refrigerator at −80 °C. The July and November samples from wild pikas were named the JW and NW groups, respectively. All procedures were approved by the Animal Care and Use Committee of Northwest Institute of Plateau Biology, Chinese Academy of Sciences.

### 4.2. Experimental Design

Forty wild pikas, twenty in July and twenty in November, were transported to the laboratory. The pikas were individually housed in feeding boxes with wood chips as nest material and free access to water and food (rabbit maintenance feed; KEAO XIELI Feed Co., Ltd., Beijing, China). The temperature in the laboratory was controlled at 20 °C ± 3 °C with 20% ± 10% humidity and natural light. The pikas were housed for 20 weeks in July and 2 weeks in November. Thereafter, 10 randomly selected individuals from July and November were euthanized and dissected on a sterile table to collect their feces, as well as WAT and BAT from the scapular region. The July and November samples from captive pikas were named the JC and NC groups, respectively. Subsequently, SW was added to the pika diet in July and November for 4 weeks. The body mass of pikas was measured at the initial point and end of each stage. Finally, all pikas were euthanized and dissected on a sterile table to collect their feces, as well as WAT and BAT from the scapular region. The July and November samples from pikas fed the SW diet were named the JSW and NSW groups, respectively. Owing to the accidental death of pikas during husbandry, nine samples were collected from the JSW group and seven from the NSW group.

### 4.3. SW Extraction

SW was extracted from *O. ochrocephala* harvested near the sampling site using an ultrasonic chloroform extraction method with a final purity of approximately 93% [68]. Thereafter, SW was dissolved in a solution with a concentration of 0.1 mg/mL in distilled water and sprayed uniformly on quantitative rabbit chow. The SW dosage was determined based on the content of SW in plants ingested by pikas under field conditions [27]. Thus, 0.1 mg SW was finally added to each pika’s diet.

### 4.4. The Amplicon Sequencing Analysis of the 16S rRNA Gene

Total DNA was extracted from fecal samples using the QIAamp DNA Stool Mini Kit (Qiagen, Dusseldorf, Germany) according to the manufacturer’s protocol. The DNA concentration was determined using a NanoDrop ND-1000 system (Thermo Fisher Scientific, Waltham, MA, USA). Universal primers 341F (5′-CCTAYGGGRBGCASCAG-3′) and 806R (5′-GGACTACNNGGGTATCTAAT-3′) were used to amplify the V3–V4 regions of 16S rDNA. The TruSeq^®^ DNA PCR-Free Sample Preparation Kit (Illumina, San Diego, CA, USA) was used to generate sequencing libraries. Finally, the library was sequenced on the HiSeq2500 platform (Illumina).

The resulting PE reads from Illumina sequencing were split into samples. Paired-end reads were quality-controlled and filtered based on sequencing quality. Splicing was subsequently performed based on the overlapping relationships between paired-end reads. Representative amplicon sequence variant (ASV) sequences and abundance information were obtained after noise reduction by DADA2 [69] within Quantitative Insight Into Microbial Ecology 2 (QIIME2, http://QIIME2.org/index.html, accessed on 1 February 2020). Finally, the representative sequences and abundances of the ASVs were searched against the Silva reference database (https://www.arb-silva.de, accessed on 27 August 2020). Functional categories were calculated with PICRUSt2 using the Kyoto Encyclopedia of Genes and Genomes (KEGG) database (https://www.kegg.jp, accessed on 1 April 2022).

### 4.5. WAT Morphology

The WAT of pikas was fixed in 4% paraformaldehyde solution overnight at 4 °C. Following dehydration, samples were embedded in paraffin and sections of approximately 5 µm thickness were mounted onto glass slides. Hematoxylin staining solution was added dropwise to the slides, stained for 30 s, and slides were rinsed with tap water for another 30 s. Eosin staining solution was added dropwise, stained for 10 s, and slides were rinsed with tap water for 30 s. The slides were blocked with neutral gum after drying. The sections were observed under a light microscope (Nikon ECLIPSE E200), and images were selected from clear and clean fields with complete cell morphology. The cells were marked using Image-Pro Plus software v6.0, and the diameters and cross-sectional areas of the cells were measured.

### 4.6. Statistical Analysis

Student’s *t*-tests were used to examine group differences in body mass, body mass gain, BAT mass, BAT mass/body mass, and the diameter and cross-sectional area of WAT. PCoA based on the Bray–Curtis distance revealed the separation of gut microbial communities and functions of plateau pikas between groups. PERMANOVA was used to determine whether the differences between groups were significant. Heatmaps were used to display the sample distribution of bacterial and functional abundance. Group differences were assessed using the Mann–Whitney U test. Spearman’s correlation of bacteria at the species level, with a relative abundance > 0.01% in each group, was analyzed using the “psych” package [70] in R (https://www.r-project.org, accessed on 29 February 2020). The results were presented as a network using Gephi v0.9.2 [71]. NTI values were calculated using the “picante” package [72] in R to identify the gut microbiota assembly process. The Kruskal–Wallis test followed by multiple comparison post hoc tests was used to analyze differences in alpha diversity, Bray–Curtis distance, node-level network parameters, and NTI values between the groups. RDA was performed using the “vegan” package [73] in R to reveal the relationship between BAT mass and the gut microbiota.

## 5. Conclusions

In the present study, we found that SW inhibited body mass gain and contributed to an increase in BAT mass and WAT browning in pikas. The 16S rDNA sequencing revealed that the alpha diversity of the gut microbiota was significantly reduced and the community structure of the gut microbiota was significantly altered in captive pikas. However, the addition of SW to the diet significantly increased the alpha diversity of the gut microbiota. SW significantly increased the relative abundance of *Akkermansia*, *Prevotella* and *unclassified_f__Lachnospiraceae*, along with enhancing the complexity of the microbial co-occurrence network structure, which decreased in the gut of captive pikas. The functional profiles showed that SW significantly decreased the relative abundances of energy metabolism, lipid metabolism, and glycan biosynthesis and metabolism, which were enriched in captive pikas. Furthermore, SW decreased the deterministic processes of gut microbiota assembly in July and increased them in November. Finally, the genera *Prevotella* and *unclassified_f__Prevotellaceae* were shown to have a positive correlation with BAT mass. Our results highlighted the role of PSCs in promoting WAT browning via gut microbiota modulation in small mammals.

## Data Availability

All raw sequences in this study were submitted to the National Center for Biotechnology Information database with the accession number PRJNA613933.

## Supplementary Materials

The following supporting information can be downloaded at: https://www.mdpi.com/article/10.3390/ijms242417420/s1.

## References

[1] Tanaka M. (2020). Improving obesity and blood pressure. Hypertens. Res..

[2] Lu X.Q., Jin Y.X., Li D.X., Zhang J.X., Han J.Y., Li Y. (2022). Multidisciplinary Progress in Obesity Research. Genes.

[3] Klein S., Gastaldelli A., Yki-Järvinen H., Scherer P.E. (2022). Why does obesity cause diabetes?. Cell Metab..

[4] WHO (2021). Obesity and Overweight. https://www.who.int/news-room/fact-sheets/detail/obesity-and-overweight.

[5] WHO (2021). Facts in Pictures: Obesity. https://www.who.int/news-room/facts-in-pictures/detail/6-facts-on-obesity.

[6] Landovská P., Karbanová M. (2023). Social costs of obesity in the Czech Republic. Eur. J. Health Econ..

[7] Ataey A., Jafarvand E., Adham D., Moradi-Asl E. (2020). The Relationship Between Obesity, Overweight, and the Human Development Index in World Health Organization Eastern Mediterranean Region Countries. J. Prev. Med. Public Health.

[8] Dobbs R., Sawers C., Thompson F., Manyika J., Woetzel J.R., Child P., McKenna S., Spatharou A. (2014). Overcoming Obesity: An Initial Economic Analysis.

[9] Calderon-Dominguez M., Mir J.F., Fucho R., Weber M., Serra D., Herrero L. (2016). Fatty acid metabolism and the basis of brown adipose tissue function. Adipocyte.

[10] Schrauwen P., van Marken Lichtenbelt W.D., Spiegelman B.M. (2015). The future of brown adipose tissues in the treatment of type 2 diabetes. Diabetologia.

[11] Jeremic N., Chaturvedi P., Tyagi S.C. (2017). Browning of White Fat: Novel Insight into Factors, Mechanisms, and Therapeutics. J. Cell. Physiol..

[12] Bartelt A., Heeren J. (2013). Adipose tissue browning and metabolic health. Nat. Rev. Endocrinol..

[13] Xue H., Wang Z., Hua Y., Ke S., Wang Y., Zhang J., Pan Y.-H., Huang W., Irwin D.M., Zhang S. (2018). Molecular signatures and functional analysis of beige adipocytes induced from in vivo intra-abdominal adipocytes. Sci. Adv..

[14] Bai Z.Z., Tana W.R., Liu S., Han S.R., Chen L., McClain D., Ge R.L. (2015). Intermittent cold exposure results in visceral adipose tissue “browning” in the plateau pika (*Ochotona curzoniae*). Comp. Biochem. Physiol. Part A.

[15] Gao Y., Qimuge N.R., Qin J., Cai R., Li X., Chu G.Y., Pang W.J., Yang G.S. (2018). Acute and chronic cold exposure differentially affects the browning of porcine white adipose tissue. Animal.

[16] Li G., Xie C., Lu S., Nichols R.G., Tian Y., Li L., Patel D., Ma Y., Brocker C.N., Yan T. (2017). Intermittent Fasting Promotes White Adipose Browning and Decreases Obesity by Shaping the Gut Microbiota. Cell Metab..

[17] Lone J., Choi J.H., Kim S.W., Yun J.W. (2016). Curcumin induces brown fat-like phenotype in 3T3-L1 and primary white adipocytes. J. Nutr. Biochem..

[18] Azhar Y., Parmar A., Miller C.N., Samuels J.S., Rayalam S. (2016). Phytochemicals as novel agents for the induction of browning in white adipose tissue. Nutr. Metab..

[19] Bäckhed F., Ding H., Wang T., Hooper L.V., Koh G.Y., Nagy A., Semenkovich C.F., Gordon J.I. (2004). The gut microbiota as an environmental factor that regulates fat storage. Proc. Natl. Acad. Sci. USA.

[20] Du L., Lei X., Wang J., Wang L., Zhong Q., Fang X., Li P., Du B., Wang Y., Liao Z. (2021). Lipopolysaccharides derived from gram-negative bacterial pool of human gut microbiota promote inflammation and obesity development. Int. Rev. Immunol..

[21] Cheng Z., Zhang L., Yang L., Chu H. (2022). The critical role of gut microbiota in obesity. Front. Endocrinol..

[22] Zuojian F., Changlin Z. (1985). Studies on the pikas (genus: *Ochotona*) of China—Taxonomic notes and distribution. Acta Theriol. Sin..

[23] An Z., Wei L., Xu B., Wang Z., Gao C., Li J., Wei L., Qi D., Shi P., Zhang T. (2022). A homotetrameric hemoglobin expressed in alveolar epithelial cells increases blood oxygenation in high-altitude plateau pika (*Ochotona curzoniae*). Cell Rep..

[24] Speakman J.R., Chi Q., Ołdakowski Ł., Fu H., Fletcher Q.E., Hambly C., Togo J., Liu X., Piertney S.B., Wang X. (2021). Surviving winter on the Qinghai-Tibetan Plateau: Pikas suppress energy demands and exploit yak feces to survive winter. Proc. Natl. Acad. Sci. USA.

[25] Wang J.-M., Zhang Y.-M., Wang D.-H. (2006). Seasonal thermogenesis and body mass regulation in plateau pikas (*Ochotona curzoniae*). Oecologia.

[26] Lu H., Wang S.S., Wang W.L., Zhang L., Zhao B.Y. (2014). Effect of swainsonine in *Oxytropis kansuensis* on Golgi alpha-mannosidase II expression in the brain tissues of Sprague-Dawley rats. J. Agric. Food Chem..

[27] Jiang Z., Xia W. (1985). Utilization of the food resources by plateau pika. Acta Theriol. Sin..

[28] Fan C., Zhang L.Z., Fu H.B., Liu C.F., Li W.J., Cheng Q., Zhang H., Jia S.A., Zhang Y.M. (2020). Enterotypes of the Gut Microbial Community and Their Response to Plant Secondary Compounds in Plateau Pikas. Microorganisms.

[29] Ren S., Fan C., Zhang L., Tang X., Fu H., Liu C., Jia S., Zhang Y. (2021). The plant secondary compound swainsonine reshapes gut microbiota in plateau pikas (*Ochotona curzoniae*). Appl. Microbiol. Biotechnol..

[30] Bhardwaj M., Yadav P., Vashishth D., Sharma K., Kumar A., Chahal J., Dalal S., Kataria S.K. (2021). A review on obesity management through natural compounds and a green nanomedicine-based approach. Molecules.

[31] Mir S.A., Shah M.A., Ganai S.A., Ahmad T., Gani M. (2019). Understanding the role of active components from plant sources in obesity management. J. Saudi Soc. Agric. Sci..

[32] Yang X.D., Ge X.C., Jiang S.Y., Yang Y.Y. (2022). Potential lipolytic regulators derived from natural products as effective approaches to treat obesity. Front. Endocrinol..

[33] Song D., Cheng L., Zhang X., Wu Z., Zheng X. (2019). The modulatory effect and the mechanism of flavonoids on obesity. J. Food Biochem..

[34] Lipina C., Hundal H.S. (2014). Carnosic acid stimulates glucose uptake in skeletal muscle cells via a PME-1/PP2A/PKB signalling axis. Cell Signal.

[35] Mougios V., Ring S., Petridou A., Nikolaidis M.G. (2003). Duration of coffee- and exercise-induced changes in the fatty acid profile of human serum. J. Appl. Physiol..

[36] Diepvens K., Westerterp K.R., Westerterp-Plantenga M.S. (2007). Obesity and thermogenesis related to the consumption of caffeine, ephedrine, capsaicin, and green tea. Am. J. Physiol. Regul. Integr. Comp. Physiol..

[37] Zhang X., Zhang Q.X., Wang X., Zhang L., Qu W., Bao B., Liu C.A., Liu J. (2016). Dietary luteolin activates browning and thermogenesis in mice through an AMPK/PGC1alpha pathway-mediated mechanism. Int. J. Obes..

[38] Milton-Laskibar I., Gomez-Zorita S., Arias N., Romo-Miguel N., Gonzalez M., Fernandez-Quintela A., Portillo M.P. (2020). Effects of resveratrol and its derivative pterostilbene on brown adipose tissue thermogenic activation and on white adipose tissue browning process. J. Physiol. Biochem..

[39] Hale V.L., Tan C.L., Niu K., Yang Y., Zhang Q., Knight R., Amato K.R. (2019). Gut microbiota in wild and captive Guizhou snub-nosed monkeys, *Rhinopithecus brelichi*. Am. J. Primatol..

[40] Gao H., Chi X., Qin W., Wang L., Song P., Cai Z., Zhang J., Zhang T. (2019). Comparison of the gut microbiota composition between the wild and captive Tibetan wild ass (*Equus kiang*). J. Appl. Microbiol..

[41] Li Y., Hu X., Yang S., Zhou J., Zhang T., Qi L., Sun X., Fan M., Xu S., Cha M. (2017). Comparative Analysis of the Gut Microbiota Composition between Captive and Wild Forest Musk Deer. Front. Microbiol..

[42] Li Y., Yan Y., Fu H., Jin S., He S., Wang Z., Dong G., Li B., Guo S. (2023). Does diet or macronutrients intake drive the structure and function of gut microbiota?. Front. Microbiol..

[43] Zhang M., Yang X.J. (2016). Effects of a high fat diet on intestinal microbiota and gastrointestinal diseases. World J. Gastroenterol..

[44] Domínguez-Avila J.A., Villa-Rodriguez J.A., Montiel-Herrera M., Pacheco-Ordaz R., Roopchand D.E., Venema K., González-Aguilar G.A. (2021). Phenolic Compounds Promote Diversity of Gut Microbiota and Maintain Colonic Health. Dig. Dis. Sci..

[45] Kohl K.D., Dearing M.D. (2012). Experience matters: Prior exposure to plant toxins enhances diversity of gut microbes in herbivores. Ecol. Lett..

[46] Lozupone C.A., Stombaugh J.I., Gordon J.I., Jansson J.K., Knight R. (2012). Diversity, stability and resilience of the human gut microbiota. Nature.

[47] Liao W., Yin X., Li Q., Zhang H., Liu Z., Zheng X., Zheng L., Feng X. (2018). Resveratrol-Induced White Adipose Tissue Browning in Obese Mice by Remodeling Fecal Microbiota. Molecules.

[48] Han X., Guo J., Yin M., Liu Y., You Y., Zhan J., Huang W. (2020). Grape Extract Activates Brown Adipose Tissue Through Pathway Involving the Regulation of Gut Microbiota and Bile Acid. Mol. Nutr. Food Res..

[49] Li J., Zhang L., Li Y., Wu Y., Wu T., Feng H., Xu Z., Liu Y., Ruan Z., Zhou S. (2020). Puerarin improves intestinal barrier function through enhancing goblet cells and mucus barrier. J. Funct. Foods.

[50] Bi Y., Yang C., Diao Q., Tu Y. (2017). Effects of dietary supplementation with two alternatives to antibiotics on intestinal microbiota of preweaned calves challenged with *Escherichia coli* K99. Sci. Rep..

[51] Joch M., Mrázek J., Skřivanová E., Čermák L., Marounek M. (2018). Effects of pure plant secondary metabolites on methane production, rumen fermentation and rumen bacteria populations in vitro. J. Anim. Physiol. Anim. Nutr..

[52] Li G.L., Li J., Kohl K.D., Yin B.F., Wei W.H., Wan X.R., Zhu B.L., Zhang Z.B. (2019). Dietary shifts influenced by livestock grazing shape the gut microbiota composition and co-occurrence networks in a local rodent species. J. Anim. Ecol..

[53] Zhang S., Shu J., Xue H., Zhang W., Zhang Y., Liu Y., Fang L., Wang Y., Wang H., Heck M. (2020). The Gut Microbiota in Camellia Weevils Are Influenced by Plant Secondary Metabolites and Contribute to Saponin Degradation. mSystems.

[54] García-Amado M., Michelangeli F., Gueneau P., Perez M., Domínguez-Bello M. (2007). Bacterial detoxification of saponins in the crop of the avian foregut fermenter *Opisthocomus hoazin*. J. Anim. Feed Sci..

[55] Santolini M., Barabási A.L. (2018). Predicting perturbation patterns from the topology of biological networks. Proc. Natl. Acad. Sci. USA.

[56] Fan C., Zhang L., Jia S., Tang X., Fu H., Li W., Liu C., Zhang H., Cheng Q., Zhang Y. (2022). Seasonal variations in the composition and functional profiles of gut microbiota reflect dietary changes in plateau pikas. Integr. Zool..

[57] Martínez-Mota R., Kohl K.D., Orr T.J., Dearing M.D. (2020). Natural diets promote retention of the native gut microbiota in captive rodents. ISME J..

[58] Li B., Gao H.M., Song P.F., Liang C.B., Jiang F., Xu B., Liu D.X., Zhang T.Z. (2022). Captivity Shifts Gut Microbiota Communities in White-Lipped Deer (*Cervus albirostris*). Animals.

[59] Maurice C.F., Cl Knowles S., Ladau J., Pollard K.S., Fenton A., Pedersen A.B., Turnbaugh P.J. (2015). Marked seasonal variation in the wild mouse gut microbiota. ISME J..

[60] Xia T., Yao Y., Wang C., Dong M., Wu Y., Li D., Xie M., Ni Q., Zhang M., Xu H. (2021). Seasonal dynamics of gut microbiota in a cohort of wild Tibetan macaques (*Macaca thibetana*) in western China. Glob. Ecol. Conserv..

[61] Gacesa R., Kurilshikov A., Vich Vila A., Sinha T., Klaassen M.A.Y., Bolte L.A., Andreu-Sanchez S., Chen L., Collij V., Hu S. (2022). Environmental factors shaping the gut microbiome in a Dutch population. Nature.

[62] Bajinka O., Darboe A., Tan Y., Abdelhalim K.A., Cham L.B. (2020). Gut microbiota and the human gut physiological changes. Ann. Microbiol..

[63] Esquivel-Elizondo S., Ilhan Z.E., Garcia-Pena E.I., Krajmalnik-Brown R. (2017). Insights into Butyrate Production in a Controlled Fermentation System via Gene Predictions. mSystems.

[64] Shah H.N., Collins D.M. (1990). *Prevotella*, a new genus to include *Bacteroides melaninogenicus* and related species formerly classified in the genus *Bacteroides*. Int. J. Syst. Bacteriol..

[65] den Besten G., van Eunen K., Groen A.K., Venema K., Reijngoud D.-J., Bakker B.M. (2013). The role of short-chain fatty acids in the interplay between diet, gut microbiota, and host energy metabolism. J. Lipid Res..

[66] Hu J., Kyrou I., Tan B.K., Dimitriadis G.K., Ramanjaneya M., Tripathi G., Patel V., James S., Kawan M., Chen J. (2016). Short-Chain Fatty Acid Acetate Stimulates Adipogenesis and Mitochondrial Biogenesis via GPR43 in Brown Adipocytes. Endocrinology.

[67] Hui S., Liu Y., Huang L., Zheng L., Zhou M., Lang H., Wang X., Yi L., Mi M. (2020). Resveratrol enhances brown adipose tissue activity and white adipose tissue browning in part by regulating bile acid metabolism via gut microbiota remodeling. Int. J. Obes..

[68] Liu Z., Zhao X., Yu Y., Wang J. (2006). Preliminary study on improving the extraction technology of the Swainsonine from *Oxytropis kansuensis*. J. Northwest Sci-Tech Univ. Agric. For. (Nat. Sci. Ed.).

[69] Callahan B.J., McMurdie P.J., Rosen M.J., Han A.W., Johnson A.J.A., Holmes S.P. (2016). DADA2: High-resolution sample inference from Illumina amplicon data. Nat. Methods.

[70] Revelle W.R. (2017). Psych: Procedures for Personality and Psychological Research. https://CRAN.R-project.org/package=psych.

[71] Jacomy M., Venturini T., Heymann S., Bastian M. (2014). ForceAtlas2, a continuous graph layout algorithm for handy network visualization designed for the Gephi software. PLoS ONE.

[72] Kembel S.W., Cowan P.D., Helmus M.R., Cornwell W.K., Morlon H., Ackerly D.D., Blomberg S.P., Webb C.O. (2010). Picante: R tools for integrating phylogenies and ecology. Bioinformatics.

[73] Dixon P. (2003). VEGAN, a package of R functions for community ecology. J. Veg. Sci..

