# Supplementary material for: Plant Secondary Compounds Promote White Adipose Tissue Browning via Modulation of the Gut Microbiota in Small Mammals
Source: Int J Mol Sci. 2023 Dec 13;24(24):17420. doi: 10.3390/ijms242417420 (PMC10743627; doi:10.3390/ijms242417420)
Supplement: Supplementary file 1 [file ijms-24-17420-s001.zip › ijms-2749153-supplementary.pdf]

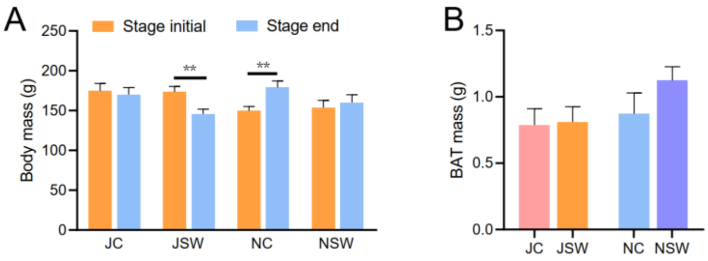

Figure S1. Changes in body mass and BAT mass of pikas. (**A**) Body mass, (**B**) BAT mass.

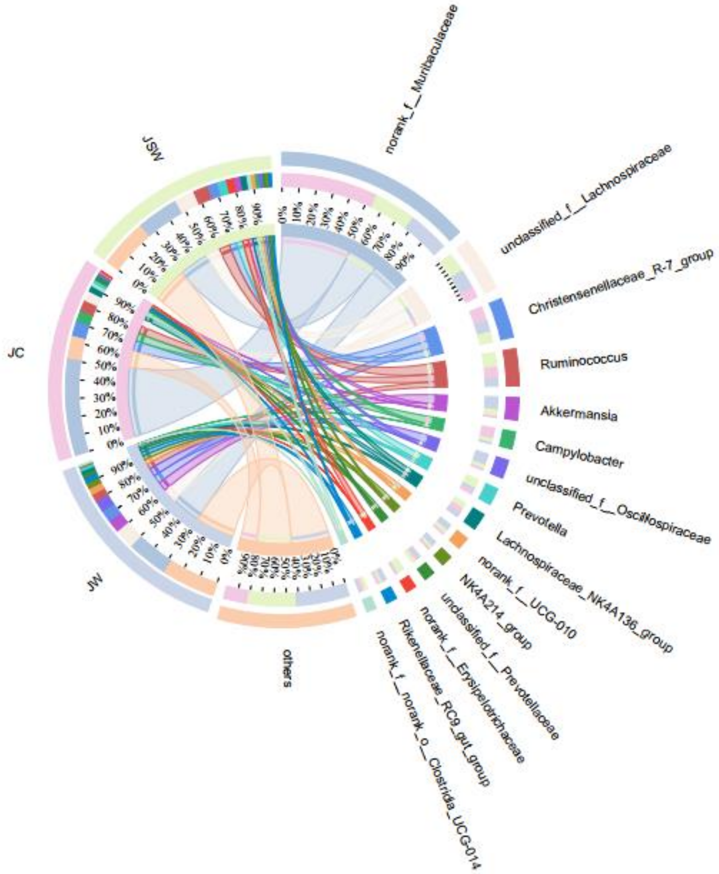

Figure S2. Pika gut bacteria composition at the genus level in July.

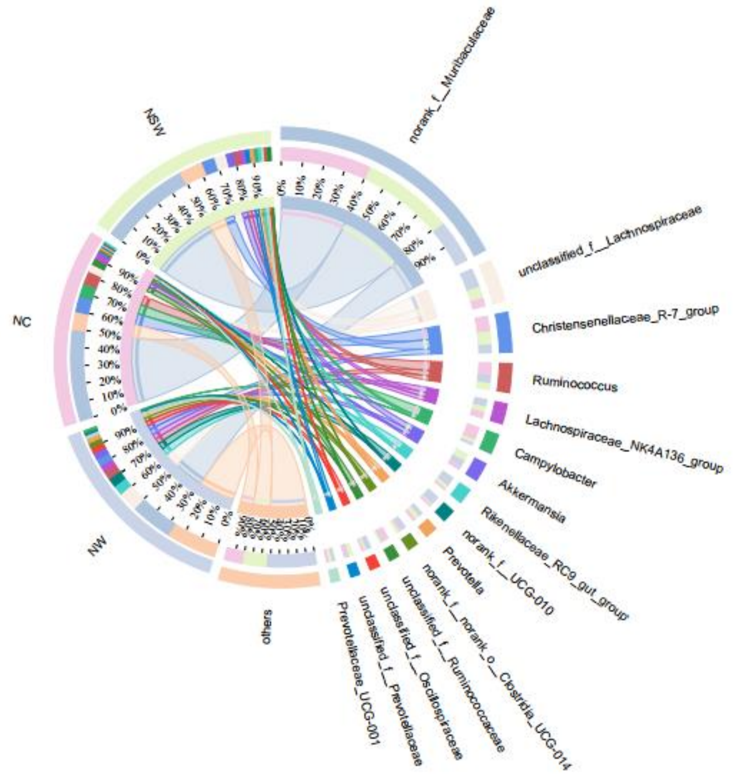

Figure S3. Pika gut bacteria composition at the genus level in November.

Table S1. PERMANOVA analysis based on Bray-Curtis distance compared the gut microbiota of pikas between different groups.

	PERMANOVA		
	F	R^2	p
JW vs JC	25.570	0.587	0.001
JW vs JSW	7.263	0.299	0.001
JC vs JSW	25.266	0.598	0.001
NW vs NC	41.809	0.699	0.001
NW vs NSW	18.651	0.554	0.001
NC vs NSW	6.348	0.297	0.001

Table S2. Results of the Mann-Whitney U test comparing the abundance of dominate bacterial taxa between plateau pikas in the JW and JC groups, and JW and JSW groups.

Taxa	JW vs JC		JW vs JSW	
	Z	p	Z	p
Phylum				
Firmicutes	**3.099**	**0.001**	-1.061	0.315
Bacteroidetes	**-3.250**	**< 0.001**	0.653	0.549
Verrucomicrobiota	**3.780**	**< 0.001**	1.715	0.095
Campilobacterota	**-3.780**	**< 0.001**	**-3.266**	**< 0.001**
Actinobacteriota	**3.704**	**< 0.001**	**3.593**	**< 0.001**
Spirochaetota	**2.873**	**0.003**	-0.898	0.400
Proteobacteria	-1.663	0.105	**-2.449**	**0.013**
Cyanobacteria	**2.117**	**0.035**	-1.715	0.095
Desulfobacterota	1.739	0.089	0.572	0.604
Patescibacteria	**-3.476**	**< 0.001**	**-3.768**	**< 0.001**
Genus				
norank_f__Muribaculaceae	**-3.780**	**< 0.001**	0.327	0.780
unclassified_f__Lachnospiraceae	**3.024**	**0.002**	-1.306	0.211
Christensenellaceae_R−7_group	-1.587	0.123	0.163	0.906
Ruminococcus	-1.965	0.052	**-2.613**	**0.008**
Akkermansia	**3.780**	**< 0.001**	1.715	0.095
Campylobacter	**-3.780**	**< 0.001**	**-3.429**	**< 0.001**
unclassified_f__Oscillospiraceae	**3.780**	**< 0.001**	**3.674**	**< 0.001**
Lachnospiraceae_NK4A136_group	**-2.646**	**0.007**	**-2.858**	**0.003**
Prevotella	0.227	0.853	**-2.613**	**0.008**
norank_f__UCG−010	**3.780**	**< 0.001**	**2.286**	**0.022**
NK4A214_group	**3.780**	**< 0.001**	**3.021**	**0.001**
unclassified_f__Prevotellaceae	1.361	0.190	1.715	0.095
Rikenellaceae_RC9_gut_group	**3.326**	**< 0.001**	**3.266**	**< 0.001**
norank_f__Erysipelotrichaceae	1.210	0.247	-3.186	0.001
norank_f__norank_o__Clostridia_UCG−014	**-3.780**	**< 0.001**	**-3.674**	**< 0.001**

Significant effects are in bold.

Table S3. Results of the Mann-Whitney U test comparing the abundance of dominate bacterial taxa between plateau pikas in the NW and NC groups, and NW and NSW groups.

Taxa	NW vs NC		NW vs NSW	
	Z	p	Z	p
Phylum				
Bacteroidetes	**-3.628**	**< 0.001**	**-3.416**	**< 0.001**
Firmicutes	**3.780**	**< 0.001**	**3.416**	**< 0.001**
Campilobacterota	**-3.780**	**< 0.001**	-1.464	0.161
Verrucomicrobiota	**3.780**	**< 0.001**	-0.098	0.962
Actinobacteriota	1.512	0.143	**2.635**	**0.007**
Proteobacteria	**3.024**	**0.002**	**2.147**	**0.033**
Spirochaetota	1.512	0.143	-1.757	0.088
Patescibacteria	-0.605	0.579	1.464	0.161
Cyanobacteria	**3.099**	**0.001**	0.098	1.000
Desulfobacterota	**3.780**	**< 0.001**	3.416	**< 0.001**
Genus				
norank_f__Muribaculaceae	**-3.780**	**< 0.001**	**-3.318**	**< 0.001**
unclassified_f__Lachnospiraceae	**3.628**	**< 0.001**	**2.733**	**0.005**
Christensenellaceae_R−7_group	**-3.477**	**< 0.001**	**-2.928**	**0.002**
Ruminococcus	-1.209	0.247	1.269	0.230
Campylobacter	**-3.780**	**< 0.001**	-1.952	0.055
Lachnospiraceae_NK4A136_group	**2.041**	**0.043**	1.366	0.193
Akkermansia	**3.780**	**< 0.001**	-0.098	0.962
Rikenellaceae_RC9_gut_group	**3.780**	**< 0.001**	**2.928**	**0.002**
norank_f__UCG−010	**3.780**	**< 0.001**	**3.416**	**< 0.001**
Prevotella	**3.326**	**< 0.001**	-0.390	0.740
norank_f__norank_o__Clostridia_UCG−014	**3.250**	**< 0.001**	**3.220**	**< 0.001**
unclassified_f__Ruminococcaceae	**-3.704**	**< 0.001**	1.659	0.109
unclassified_f__Oscillospiraceae	**3.780**	**< 0.001**	**3.220**	**< 0.001**
Prevotellaceae_UCG−001	**3.326**	**< 0.001**	-0.390	0.740
unclassified_f__Prevotellaceae	1.436	0.165	**-3.318**	**< 0.001**

Significant effects are in bold.

Table S4. Network indices of pika gut microbiota.

Network indices	JW	JC	JSW	NW	NC	NSW
Total nodes	66	68	69	68	69	69
Total links	333	251	422	355	238	329
Positive links	215	204	354	258	176	268
Negative links	118	47	68	97	62	61
Average degree	10.091	7.382	12.232	10.441	6.899	9.536
Density	0.155	0.110	0.180	0.156	0.101	0.140
Average clustering coefficient	0.643	0.561	0.622	0.612	0.569	0.697
Average path length	2.942	3.159	2.562	2.688	3.628	2.958

Table S5. PERMANOVA analysis based on Bray-Curtis distance compared the function profiles of pika gut microbiota between different groups.

	PERMANOVA		
	F	R^2	p
JW vs JC	19.015	0.214	0.002
JW vs JSW	5.596	0.248	0.006
JC v JSW	18.359	0.519	0.002
NW vs NC	16.577	0.479	0.001
NW vs NSW	10.036	0.401	0.001
NC vs NSW	8.796	0.370	0.001

Table S6. Results of the Mann-Whitney U test comparing the abundance of KEGG metabolism categories (level 2) between plateau pikas in the JW and JC groups, and JW and JSW groups.

KEGG level 2	JW vs JC		JW vs JSW	
	Z	p	Z	p
Global and overview maps	-1.890	0.063	**-2.531**	**0.010**
Carbohydrate metabolism	1.890	0.063	**-2.613**	**0.008**
Amino acid metabolism	**3.780**	**< 0.001**	**3.674**	**< 0.001**
Energy metabolism	**-3.553**	**< 0.001**	-0.163	0.905
Metabolism of cofactors and vitamins	0.076	1.000	**2.286**	**0.022**
Translation	**3.628**	**< 0.001**	**3.511**	**< 0.001**
Replication and repair	**3.780**	**< 0.001**	**3.184**	**0.001**
Membrane transport	-0.076	0.971	-1.633	0.113
Nucleotide metabolism	**2.948**	**0.002**	**2.449**	**0.013**
Signal transduction	0.680	0.529	0.082	1.000
Cellular community − prokaryotes	0.454	0.684	-0.980	0.356
Lipid metabolism	**-3.780**	**< 0.001**	-1.633	0.113
Glycan biosynthesis and metabolism	**-2.041**	**0.043**	0.653	0.549
Biosynthesis of other secondary metabolites	-1.890	0.063	-0.245	0.842
Folding, sorting and degradation	-0.680	0.529	1.388	0.182

Significant effects are in bold.

Table S7. Results of the Mann-Whitney U test comparing the abundance of KEGG metabolism categories (level 2) between plateau pikas in the NW and NC groups, and NW and NSW groups.

KEGG level 2	NW vs NC		NW vs NSW	
	Z	p	Z	p
Global and overview maps	**-3.780**	**< 0.001**	-1.171	0.270
Carbohydrate metabolism	**2.192**	**0.029**	**-2.733**	**0.005**
Amino acid metabolism	**3.780**	**< 0.001**	**3.416**	**< 0.001**
Energy metabolism	**-3.780**	**< 0.001**	**-2.342**	**0.019**
Metabolism of cofactors and vitamins	-0.832	0.436	**-2.049**	**0.043**
Translation	**-2.041**	**0.043**	1.269	0.230
Replication and repair	1.512	0.143	1.073	0.315
Membrane transport	1.209	0.247	1.366	0.193
Nucleotide metabolism	1.361	0.190	-1.757	0.088
Signal transduction	**2.797**	**0.004**	**2.733**	**0.005**
Cellular community − prokaryotes	**2.948**	**0.002**	**2.440**	**0.014**
Lipid metabolism	-1.512	0.143	-1.366	0.193
Glycan biosynthesis and metabolism	**-3.477**	**< 0.001**	**-3.416**	**< 0.001**
Biosynthesis of other secondary metabolites	**-2.570**	**0.009**	**-3.318**	**< 0.001**
Folding, sorting and degradation	**-2.495**	**0.011**	0.586	0.601

Significant effects are in bold.
